# A novel co-operative mechanism linking TGFβ and Lyn kinase activation to imatinib resistance in chronic myeloid leukaemia cells

**DOI:** 10.18632/oncotarget.500

**Published:** 2012-05-23

**Authors:** Paul G. Smith, Hideo Tanaka, Andrew Chantry

**Affiliations:** ^1^ School of Biological Sciences, University of East Anglia, Norwich, UK; ^2^ Department of Hematology, Hiroshima City Asa Hospital, Asakita-ku, Hiroshima, Japan.

**Keywords:** Transforming growth factor-β, Smads, ubiquitin, c-cbl, Lyn, leukaemia

## Abstract

The advent of a mechanism specific inhibitor imatinib, targeting Bcr-Abl kinase, has paved the way for new treatment strategies in chronic myeloid leukaemia (CML). However, resistance to imatinib is common in patients and has recently been linked to both transforming growth factor-β (TGFβ) and elevated Lyn kinase activity, although molecular mechanisms remain largely unknown. Here, using leukaemic MYL cell lines derived from CML patients, we show that TGFβ plays a key role in imatinib-resistance via direct effects on Lyn ubiquitination and turnover that results in bursts of Lyn kinase activity, and identify c-cbl is a candidate E3 ubiquitin ligase. Furthermore, blockade of TGFβ signalling activity with the TGFβ receptor kinase inhibitor SB431542 significantly reduces Lyn turnover and activation, and subsequently enhances imatinib-mediated CML cell death in a proteasomal-dependent manner. Collectively, our data reveals novel co-operative mechanisms in CML involving TGFβ and Lyn kinase linked to proteasome function and ubiquitination, and thus supports therapeutic approaches that target TGFβ pathway activity as a strategy for overcoming imatinib-resistance in CML.

## INTRODUCTION

Chronic myeloid leukaemia (CML) is a disease of haematopoietic stem cells and originates from a specific chromosomal transfer event that generates a hybrid Bcr-Abl oncoprotein with tyrosine-kinase activity and cell transforming capacity [1]. Recently, a Bcr-Abl kinase inhibitor (Imatinib, Gleevac or STI571) has been used to treat CML with some success [2]. However, patients become resistant mainly due to a small group of immature Bcr-Abl +ve stem cells in the bone marrow that have stopped dividing, and become refractory to the effects of an inhibitor which is only effective against growing cells [3]. Mechanisms associated with imatinib-resistance include Bcr-Abl point mutations and increased Bcr-Abl protein expression [4, 5]. In addition, Bcr-Abl-independent mechanisms of resistance have been proposed [6], including elevated P-glycoprotein drug efflux [7], and the recruitment of other signalling cascades [8]. Most notably, several recent studies highlight an emerging role for Lyn kinase overexpression and activation in the development of imatinib-resistance in CML [9-12], and activated Lyn results in elevated levels of the anti-apoptotic protein Bcl-2, which protects CML cells from imatinib-mediated cell lethality [13]. Furthermore, in CML cells, knockdown of elevated Lyn expression by siRNA resulted in increased apoptosis and enhanced imatinib sensitivity [14].

At present, the precise molecular mechanisms responsible for increased Lyn expression and activation in CML have not been established, although it is likely to involve induction and/or re-wiring of other signalling pathways linked to haematopoietic cell function. A prime candidate in this regard is transforming growth factor-β (TGFβ), which is known to play a vital role in maintaining the growth and differentiation balance in haematopoietic cells [15]. In general, TGFβ is a robust inhibitor of committed progenitor cell function, and autocrine production of TGFβ maintains haematopoietic stem cell quiescence [16]. Several recent studies have attempted to unravel the effects TGFβ on leukaemia cell proliferation. A TGFβ/FOXO/Akt signalling axis was found to be responsible for imatinib resistance in a CML stem cell population and it was shown that pre-treatment of CML stem cells with the TGFβ receptor inhibitor LY364947 efficiently activated Akt and suppressed FOXO-induced cell cycle arrest and apoptosis [17]. Furthermore, this study demonstrated that combined treatment with LY364947 and Imatinib significantly reduced lethality in a CML mouse model and decreased stem cell frequency. In support of these data, TGFβ has also been reported as a candidate niche factor to maintain haematopoietic stem dormancy and hibernation [18]. Collectively, these data suggest that blockade of TGFβ signalling activity could potentially promote efficient eradication of residual CML stem cells, a notion that is also supported the finding that Bcr-Abl enhances TGFβ signalling activity in CML cells [19].

Interestingly, previous studies have implicated a role for TGFβ in regulating Lyn function, and TGFβ stimulation over a 30 minutes timeframe caused a rapid decrease in cellular levels in Lyn together with a decrease in Lyn kinase activity in a prostate cancer cell line [20]. Here, we investigated inter-connections between TGFβ and Lyn in the MYL cell line derived from a CML patient and find that TGFβ stimulation drives rapid ubiquitination and proteasomal turnover of Lyn. Interestingly, prolonged stimulation results in Lyn protein replenishment and a significantly elevated re-bound in the level of Lyn kinase activity. Furthermore, we identify the E3 ubiquitin ligase c-cbl as a candidate intermediate that drives TGFβ-mediated Lyn turnover/activation, and show that blocking TGFβ signalling activity reduces Lyn activity and concomitantly sensitizes CML cells to imatinib-mediated cell death. Therefore, based on this new mechanistic insight we propose that blockade of TGFβ signalling activity in combination with the Bcr-Abl inhibitor Imatinib could provide a basis for the treatment of drug-resistant CML.

## RESULTS AND DISCUSSION

### TGFβ-induced Lyn ubiquitination generates bursts of Lyn kinase activity in CML cells

**Figure 1:**
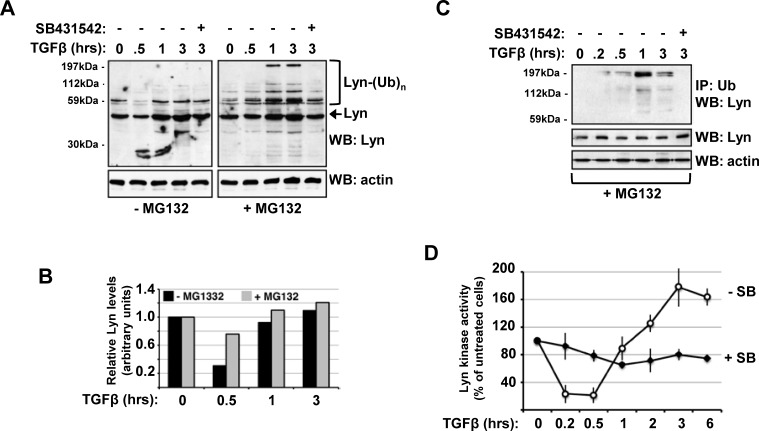
TGFβ-induced Lyn ubiquitination generates a burst of Lyn kinase activity in CML cells (A) MYL cells were serum starved overnight in 0.5% serum-containing media before treatment in the presence/absence MG132 with 1ng/ml TGFβ and/or 10μM SB431542 as indicated. Cells were lysed and Western blots probed with anti-Lyn and anti-β-Actin antibodies. (B) Blots in Figure 1A were analysed by densitometric scanning using ImageJ image analysis software, and data are representative of three experiments. (C) MYL cells were treated with 1ng/ml TGFβ and/or 10μM SB431542 in the presence of MG132 as indicated, and lysates immunoprecipitated with anti-polyubiquitin antibody were separated by Western blotting and probed with anti-Lyn or anti-actin antibodies. (D) MYL cells were treated with 1ng/ml TGFβ in the presence/absence 10μM SB431542 as indicated, lysates immunoprecipitated with anti-Lyn and immune complexes assayed for Lyn kinase assay using ATP-γP^33^ and poly(Glu-Tyr) as substrate as described in Materials and Methods. Data are representative of two independent experiments performed in triplicate, and expressed as a specific activity in relation to total lyn protein levels as determined by anti-Lyn western blotting.

Since previous studies have implicated a role for both TGFβ activity and Lyn kinase overexpression in CML, we examined whether TGF might exert a direct influence on Lyn function in MYL cells derived from a chronic phase CML patient [10]. Initially, we treated MYL cells with TGFβ and probed cellular lysates by western blotting with a Lyn antibody to monitor changes in Lyn protein levels. Unexpectedly, we found a significant decrease in the levels of Lyn after 30 minutes followed by a significant increase in Lyn following prolonged 6hours stimulation (Figure 1A). Interestingly, in the presence of the general ubiquitin-proteasome system inhibitor MG132 we observed an increase in higher molecular weight Lyn reactive bands that was not seen in the presence of the type I TGFβ receptor kinase inhibitor SB431542 (Figure 1A). Quantification also indicates that this rapid decrease and then recovery in Lyn protein levels was significantly attenuated in cells incubated with MG132 (Figure 1B).

Since the high molecular weight Lyn reactive species were seen primarily in the presence of MG132, we next tested whether they might represent ubiquitinated Lyn species. There is a clear induction of high molecular weight polyubiquitinated Lyn protein that peaks at 1hour of stimulation, and this is not seen in cells that were pre-treated with 10μM SB431542 (Figure 1C). Next, we examined whether prolonged stimulation of MYL cells with TGFβ affects Lyn protein kinase activity using a specific immune kinase assay and poly(Glu-Tyr) as substrate. Following 30minutes TGFβ stimulation, Lyn kinase activity declines rapidly but then recovers again to peak after 3hours (Figure 1D). This influence of TGFβ on Lyn kinase activity was confirmed by treatment of cells in the presence of 10μM SB431542 in which there is a slow but steady drop in Lyn activity (Figure 1D).

**Figure 2:**
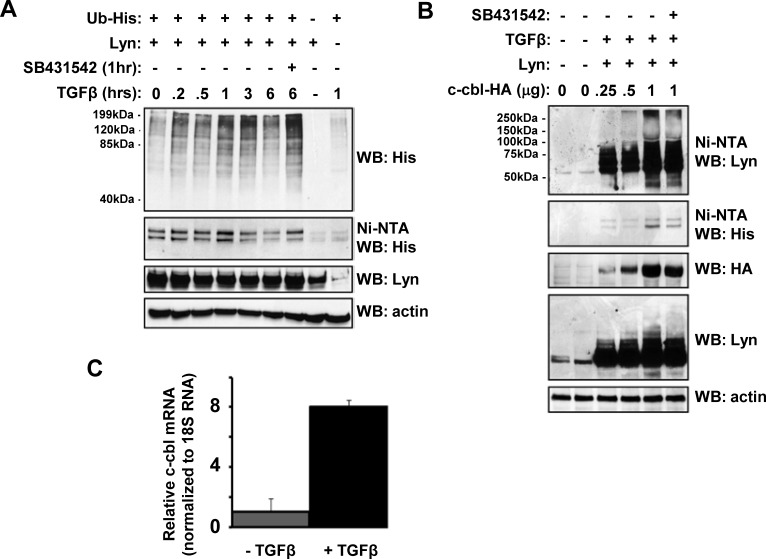
TGFβ stimulates c-cbl-dependent Lyn ubiquitination (A) TGFβ induces Lyn ubiquitination. HEK293T cells were co-transfected with expression plasmids for Lyn and His-tagged Ubiquitin. Cells were put into 2% FCS-containing media overnight before TGFβ treatment (1ng/ml) for a range of time between 0-360 minutes, or 10μM SB431532. Cell lysates were incubated overnight with nickel-NTA beads, and bound proteins eluted with Laemmli buffer. Samples were separated by SDS-PAGE and Western blots probed with anti-His, anti-Lyn or anti-actin antibodies as indicated. (B) HEK293T cells were transfected with Lyn, c-Cbl and Ub plasmids as indicated. Cells were maintained in 2% FCS media post-transfection before TGFβ (1ng/ml) or SB431542 (10μM) stimulation for 60 minutes. Lysates were incubated with Nickel-NTA beads, and bound proteins eluted with Laemmli buffer. Samples were separated by SDS-PAGE and Western blots probed with anti-His, anti-Lyn or anti-actin antibodies as indicated. (C) MYL cells were stimulated with TGFβ (1ng/ml) for 60minutes. RNA was isolated, reverse transcribed, and and 5ng cDNA utilised as template DNA in qRT-PCR using primers specific for c-cbl. Data represent triplicate samples, and are normalised against 18S RNA as an internal control.

This induction of Lyn ubiquitination seen endogenously in MYL cells was then confirmed using ectopic overexpression experiments (Figure 2A). There are a number of potential E3 Ubiquitin ligases that might connect cell surface TGFβ receptor activation to Lyn ubiquitination. Previous studies highlighted dysregulated TGFβ signalling in cbl^−/−^ cells [21], as well as oncogenic cbl mutations in several myeloid malignancies [22], and c-cbl is known to interact with Lyn and drive it Lyn poly-ubiquitination [23]. Interestingly, we find that TGFβ can stimulate cbl-mediated Lyn ubiquitination in a dose-dependent manner (Figure 2B), as well as induce c-cbl mRNA expression (Figure 2C). Therefore, we suggest that mechanistically TGFβ drives bursts of Lyn ubiquitination and turnover via inducing c-cbl transcription and expression.

### Inhibition of type TGFβ receptor kinase enhances Imatinib mediated CML cell death

**Figure 3:**
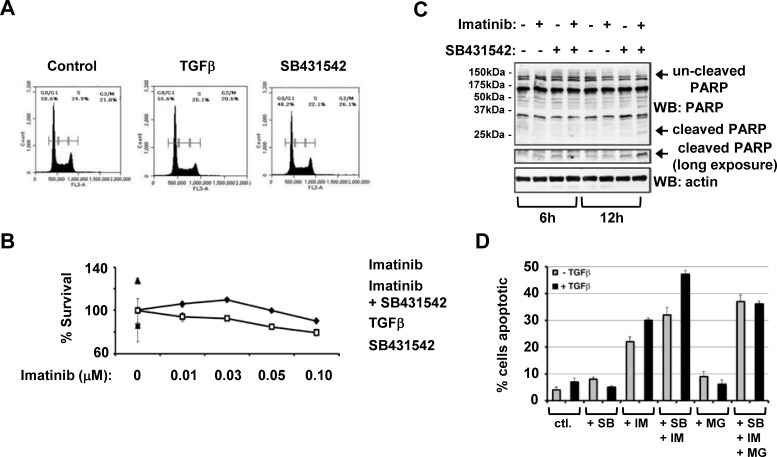
Inhibition of type TGFβ receptor kinase enhances Imatinib mediated CML cell death (A) MYL cells were synchronised with a double thymidine block, then treated with either TGFβ or SB431542 for 12 hours. Cells were fixed in 70% ethanol, incubated with propidium iodide and cell cycle status analysed by flow cytometry. Data is representative of three separate experiments and was analysed using CFlow Plus software. (B) MYL cells were serum starved in 0.5% serum containing media overnight and treated with either a range of Imatinib concentrations between 0-0.1μM or in combination with SB431542, or with TGFβ and SB431542 alone as controls. Cell viability was assessed using MTS assays. (C) MYL cells were serum starved overnight in 0.5% serum containing media before treatment with 0.3μM Imatinib, 10μM SB431542 or a combination of the two for 6 or 12 hours. Lysates were separated through SDS-PAGE, and subsequent Western blots were probed with anti-PARP or anti-actin antibodies. (D) MYL cells were pre-treated +/− 1ng/ml TGFβ followed by combination treatments with 0.3μM Imatinib, 10μM SB431542, 20μM MG132 or combinations of these as indicated, and cells were incubated for an additional 24hours and cellular apoptosis measured by annexin-V and propidium iodide staining followed by flow cytometry analysis. Data are representative of two separate experiments performed in triplicate.

Since TGFβ is able to drive bursts of Lyn kinase activity, and elevated Lyn kinase has been implicated in imatinib-resistance, we next investigated whether blockade of TGFβ signaling might influence CML cell growth and sensitivity to Imatinib. Cell Cycle analysis indicates that addition of TGFβ increases the proportion of MYL cells in G0/G1 phase by 5%, concomitant with a 5% reduction in the S phase population (Figure 3A). In the presence of SB431542 alone there is a decrease in the G0/G1 cell population and a 2% increase in cells in S phase indicative also of relatively high autocrine TGFβ activity, and an increase of almost 6% in the G2/M population compared to TGFβ treated cells (Figure 3A). Therefore, it is apparent that inhibition of TGFβ signalling in MYL cells encourages progression through the cell cycle, an important observation in the context of dual treatment approaches since imatinib is only effective against dividing cells. We then used MTS assays to assess whether combined treatment with Imatinib and SB431542 could influence MYL cell survival. In the presence of Imatinib, MYL cell survival gradually falls with increasing doses up to 0.1μM, and combined with SB431542 which in this instance blocks autocrine TGFβ signaling activity there is a significant decrease in cell survival (Figure 3B). We then examined whether SB431542 enhanced Imatinib-mediated cell death using Poly (ADP-ribose) polymerase (PARP) cleavage assays to assess cells undergoing apoptosis. There is a significant reduction in un-cleaved PARP (113kDa) in MYL cells exposed for 12 hours to both SB431542 and Imatinib combined with a clear increase in cleaved PARP at 29kDa (Figure 3C). Finally, we used an alternative assay for apoptosis based on changes in Annexin-V staining as monitored using flow cytometry. Here, cells were pre-treated in the presence/absence TGFβ for 1hour to drive Lyn turnover, and combination treatment of imatinib with SB431542 significantly enhances apoptosis compared to either imatinib or SB treatment alone (Figure 3D). Furthermore, we find that pre-treatment with MG132 attenuates the combined effect of imatinib and SB431542 implicating the ubiquitin proteasome system in this dual additive response, although this may be an indirect effect not necessarily related to TGFβ-induced Lyn ubiquitination.

In summary, we have shown for the first time direct inter-connections between TGFβ and Lyn kinase in a cell line derived from a CML patient. We find that prolonged TGFβ stimulation drives ubiquitination and rapid turnover of Lyn resulting in Lyn replenishment and bursts of Lyn kinase activity. Furthermore, we show that inhibiting endogenous TGFβ signalling reduces Lyn activity and concomitantly encourages exit from cell cycle arrest and sensitizes CML cells to imatinib-mediated cell death. Therefore, our findings highlight new mechanistic insight linked to CML disease resistance, and suggest that the use of TGFβ inhibitors in combination with Imatinib to overcome drug-resistant CML clearly warrants further consideration. Our future work will be focussed on extending our findings to a range of primary cells from imatinib-sensitive and resistant patients to confirm clinical significance, and understanding further *in vivo* mechanisms linked to CML pathology as well as characterising specific E3 ubiquitin ligases responsible for TGFβ-induced Lyn ubiquitination.

## MATERIALS AND METHODS

### Cell lines, reagents, treatments, and western blot analysis

MYL cells were maintained as described previously in RPMI supplemented with 10% Foetal Bovine Serum, 1% Penicillin Streptomycin (p/s), glutamine (200mM). HEK-293 cells were maintained in Dulbecco's modified Eagle's medium containing 10% foetal bovine serum, 1% Penicillin Streptomycin (p/s), and glutamine (200mM). Lyn cDNA cloned into a pBOS-Flag expression vector was obtained from Hiroshi Murakami (Okayama University), c-cbl expression vector from Stan Lipkowitz, National Institutes of Health, Maryland, and Ubiquitin-His from Sylvie Urbe (Liverpool University, UK). These plasmids were used for transient transfection of HEK-293 cells using LipoD transfection reagent (SignaGen Laboratories, USA). Imatinib mesylate (STI571, Gleevec) was obtained from Novartis Pharmaceuticals (Basel, Switzerland), and SB431542 (TGFβ-RI inhibitor) from Tocris Ltd. Antibodies used included anti-Lyn (New England Biolabs), anti-ubiquitin (Sigma), anti-Smad3 and anti-phospho-Smad3 (BD transduction laboratories), anti-His (Amersham Biosciences), anti-PARP (Abcam), anti-HA (Roche) and anti-β-actin (Sigma). Secondary antibodies were HRP-conjugated goat anti-rabbit or goat anti-mouse (Sigma). Cell lysis, SDS-PAGE and western blotting performed as described previously [19, 24].

### Lyn kinase assays

Lyn kinase activity was assessed by immunoprecipitation of Lyn followed by an *in vitro* kinase assay of this immune complex in the presence of ATP-γP^33^ as described previously [25].

### Ubiquitination studies

Transfected cells treated with either 20μM MG132 for 5 hours in DMEM medium containing 2% FCS and treated +/− 5ng/ml TGFβ for 1hr, washed in cold PBS, lysed in 1% v/v Igepal-630, 50mM Tris pH 8.0, 150mM NaCl, 10% v/v glycerol, 5mM EDTA 1mM NaF, 1mM Na_3_VO_4_ and protease inhibitors. Lysates were cleared by centrifugation and incubated with 0.5μg high affinity anti-HA or anti-Ub antibody and 20μl of protein-G agarose (Sigma) overnight at 4°C. Immune-complexes were harvested (2000 rpm; 30 sec), and repeatedly washed using 0.1% NP-40 LB. Immunoprecipitates were resuspended in 15μl Laemelli buffer (+10mM DTT), and analysed by Western blotting.

### Quantitative real-time qPCR

RNA was extracted using the SV Total RNA Isolation System (Promega) according to the manufacturer's instructions. To generate cDNA, 0.1μg of RNA was reverse transcribed per reaction using reverse transcriptase (Amersham) and random primers (Invitrogen) according to standard protocols, and gene expression was normalized against the housekeeping gene 18S. The reaction mix was set up as described previously [26], and cycling conditions were 2 minutes at 50°C, 10 minutes at 95°C, 15 seconds at 95°C repeated 40 times and 60°C for 1 minute. The c-Cbl primer/probes were obtained from Applied Biosytems (Hs00231981_m1), and reactions were performed using an ABI PRISM 7500 thermocycler (Applied Biosystems).

### Cell cycle analysis

Cell cycle status was assessed following the staining of cells with propidium iodide (PI) and flow cytometry analysis. Stained cells were counted on a FACScan flow cytometer (Becton Dickinson), and the data obtained was analysed using CFlow Plus as described previously [19].

### MTS and apoptosis assays

Approximately 5000 cells were seeded into 96 well plates in a volume of 100μl per well and grown overnight in 0.5% FCS-containing media. Cells were pre-treated accordingly, with SB431542 (10μM) or imatinib (10μM) prior to the addition of TGFβ. Cells were then left at 37°C for 48 hours, and 10μl of the MTS solution (Promega) was added to each well. Cells were further incubated for 3 hours before absorbance readings were taken at 490nm. When using flow cytometry to measure apoptosis, cell samples were collected by centrifugation (500g; 5min), then resupended and stained with Annexin-V and PI dye, followed by detection.
